# Supplementary material for: Are carbon emissions trading and green financial instruments synergistic? -Comprehensive quantitative research based on content analysis
Source: PLoS One. 2024 Mar 7;19(3):e0298601. doi: 10.1371/journal.pone.0298601 (PMC10919601; doi:10.1371/journal.pone.0298601)
Supplement: S1 Data — (ZIP) [file pone.0298601.s001.zip › Data/地级市Z_F工作报告.zip!/║■─╧/╓Ω╓▐╩╨/└┤╫╘╣1⁄2╓┌║┼í╛┬φ┐╦╩2╛▌═°í┐_2017.docx]

各位代表：

现在，我代表市人民政府向大会报告工作，请予审查，并请各位政协委员和其他列席人员提出意见。

一、本届政府工作回顾

市十四届人大一次会议以来，在省委、省政府和市委的坚强领导下，我们主动适应引领新常态，战胜重重困难，真抓实干，团结拼搏，圆满完成了“十二五”目标任务。

——综合实力跨上新台阶。始终保持定力，精准发力，制定实施“稳增长43条”等一系列政策措施，持续推进项目攻坚，深入开展“企业帮扶年”活动，逆势实现了稳增长，成为国务院表彰的全国20个“稳增长”先进地市之一。预计全市GDP跨上2500亿元台阶，是2012年的1.4倍；固定资产投资跨上2000亿元台阶，是2012年的2倍；一般公共预算收入突破300亿元大关，是2012年的1.5倍。人均GDP、人均一般公共预算收入等多项指标稳居全省前列。醴陵市在全国百强县排名上升至73位，攸县保持全省十强，株洲县、茶陵县、炎陵县跻身全省县域经济发展先进县行列。全面小康社会实现程度全省第二，天元区、石峰区、芦淞区、荷塘区提前达标。云龙示范区在全省两型建设示范片区中走在前列。

——转型升级实现新突破。以壮士断腕的决心关停高能耗、高污染企业，突出清水塘搬迁改造，推动有色冶炼、化工、建材等一批传统企业焕发新的生机。着力打造株洲·中国动力谷，轨道交通城、航空城、汽车城加快建设，轨道交通产业圆了株洲千亿产业梦。全市新增过百亿工业企业4家。成功获批国家自主创新示范区、“中国制造2025”试点示范城市。中国动力谷自主创新园一期建成开园。株洲高新区获批长江经济带国家转型升级示范开发区，全国排名从46位跃升至28位。园区规模工业增加值占比由52.3%提高到68%。规模工业增加值突破千亿大关，总量由全省第六前进到第三。加快建设九大服务业集聚区，电子商务、金融服务、商贸物流、文化旅游等现代服务业发展迅速，服务业对经济的贡献率由32.9%提升到49%。成功列入国家信息消费示范城市、国家电子商务示范城市、国家智慧城市试点。农业发展保持稳定，粮食生产实现“十三连丰”，农产品加工业产值占规模工业总产值比重由7.3%提高到9.3%。

——城乡建设呈现新气象。市区建成区从112平方公里扩大到138平方公里，城镇建成区从255平方公里扩大到337平方公里，城镇化率由59.1%提高到63%，顺利通过国家新型城镇化试点中期评估。湘江风光带、东城大道、万丰湖、湘江七桥、洞株路等一大批重点基础设施已经建成或正在加快建设。沪昆高铁、衡茶吉铁路和平汝、垄茶高速建成通车，公路总里程达2.4万公里，比2012年增加1800公里，实现了县县通铁路、县县通高速。两型社会建设第二阶段任务以及湘江保护和治理“一号重点工程”第一个三年行动计划全面完成，“三个基本行动”成效显著。空气质量持续改善，湘江株洲段水质优于国家Ⅲ类标准。森林覆盖率提高到61.85%，成功创建国家森林城市。建成30个省级美丽乡村示范村。成功创建全国文明城市，顺利通过国家卫生城市复审，荣获中国人居环境范例奖。

——改革开放迈出新步伐。在全省率先公布“三清单一目录”，全面取消非行政许可审批事项。推行“两集中、两到位”，建设领域投资项目审批改革经验全国推广。政府机构改革和乡镇区划调整全面完成，全市合并乡镇33个、行政村541个。事业单位分类改革平稳有序。实现财政供养人员只减不增。着力改革商事制度，市场主体迅速增加。市级政府平台公司改革积极推进。央企“三供一业”改革经验全省推广。构建全口径政府预算体系，全面公开财政预决算，全面实施“营改增”。深化农村信用社等金融领域改革，成功组建4家农商行和3家村镇银行。农林、司法、公共资源交易体制等改革取得重大进展。对外开放成效明显。引进内外资1382亿元，北汽二工厂、长城电脑等一批重大项目落户株洲。一批有实力的企业走出国门，在欧美国家建立了多个研发生产基地。

——民生事业取得新进步。大力实施“民生100”工程，集中力量办成了一大批实事。着力推进精准扶贫，四年减贫11.21万人，城区农村贫困人口全部脱贫。城乡居民养老保险实现并轨，城乡医保、低保应保尽保，覆盖城乡的社会保障体系基本建成。坚持以创业带动就业，建成湖南（株洲）就业创业指导中心，获评全国创业先进城市。“两房两棚”“两供两治”有力推进，建成3.3万套保障性住房，解决3万多户房屋发证历史遗留问题，改造农村危房2.6万户，解决66.4万农村人口安全饮水问题。在全省率先全域通过教育强县（市区）评估验收，义务教育学校全部建成合格学校。职教园6所院校入园办学。在全省率先全域破除“以药补医”，公立医院改革被国务院誉为“医改株洲模式”。国家公共文化服务体系示范区创建扎实推进，神农大剧院建成使用。荣获全国综治工作优秀地市“五连冠”，第三次捧回“长安杯”，连续六次获评“全国民族团结进步模范集体”。严格落实安全生产责任，安全生产形势平稳可控。加强食品安全监管，建成全省一流的食品检测中心。深入开展军民共建，实现全国双拥模范城市“六连冠”。不断加强国防动员工作，驻株解放军、武警部队和民兵预备役为全市经济社会发展作出了积极贡献。同时，统计、外事侨务、人防、移民、国安、质检、供销、气象、水文、广电、新闻出版、体育、档案、地方志、住房公积金、对口支援、妇女儿童、老龄、残联、贸促等工作都有新进步。

——政府治理能力得到新提升。大力推进“四型”政府建设，加快转变政府职能，建立健全“3+4”市政府决策体系，推动重大决策规范化、科学化、民主化。制定出台政府投资项目管理等制度性文件，政府权力配置和运行边界进一步规范。深入推进政务公开，监察、审计、法制等行政监督不断加强。大力推进依法行政，成为全省首批被赋予地方立法权的市州。自觉接受人大、政协和社会各界监督，人大代表建议、政协委员提案全部办复，满意度不断提升。深入开展党的群众路线教育实践活动和“三严三实”“两学一做”专题教育，严格落实全面从严治党“两个责任”，严格执行中央、省委和市委关于改进作风的各项规定，廉政建设和反腐败斗争取得积极成效，行政效能和服务水平明显提升。

各位代表，2016年是“十三五”的开局之年，我们按照年初部署，扎实推进各项工作，全市经济社会发展总体平稳、稳中有进。预计全市GDP同比增长8%，规模工业增加值增长7.2%，固定资产投资增长14%，社会消费品零售总额增长12%，一般公共预算收入增长7.7%，城乡居民收入增长8.5%。

（一）保持定力调结构，产业实力持续增强。三大动力产业增加值达190亿元。中车三大研究院、IGBT产业化项目加快建设，两个国家重点实验室成功获批。北汽株洲基地年产值预计突破百亿大关，北汽二工厂主体封顶。通用机场、千亿大道、机场大道、两机重大专项等项目加快建设。长城电脑竣工投产，攸县煤电一体化项目并网发电。大力推进国家自主创新示范区建设，出台“创新10条”“双创8条”，4个项目入选第二批制造强省重点项目。深入实施“146”工程，建成标准厂房302万平方米。建成创客空间20家，入驻创业团队213个。规模以上服务业营业收入增长23.5%。方特二期等一批项目建成运营。金融业增加值55亿元。第一产业增加值增长3.3%，农产品加工业总产值增长20%。完成65.3万亩重金属污染耕地修复治理和农作物种植结构调整。

（二）精准发力抓项目，有效投资持续扩大。积极参加“央企对接会”“沪洽周”，成功举办“湘台会”、轨道交通产业国际峰会，签约引资1760亿元，重点招商项目履约率达73.5%。开展“全市总动员、大干一百天、全力保投资”“看学议”和集中开竣工等活动，145个重点项目开工建设、59个竣工达效，投资突破千亿大关。开辟企业直接融资新渠道，开通全省首家市州股权交易分所。获批国家专项建设基金60.5亿元、企业债券66.1亿元。新增社会融资515.7亿元，其中直接融资占比76.5%，银行存贷款余额分别达2813亿元、1312亿元。完成集体土地征拆3.6万亩、国有土地房屋征收41万平方米，项目用地有效保障。

（三）齐心协力促统筹，城乡面貌持续改观。启动实施交通畅通、城市绿荫、旧城提质、碧水蓝天“四大行动”。湘江六桥、长株潭城际铁路、西站南路、东环北路、湘芸路等项目建成通车。株洲西客运枢纽站启动建设，湘江大道、环保大道、铁东路、湘江河东段综合治理等项目加快建设。建成街旁游园、社区游园10个。改造城市棚户区2万余户、农村危房近9000户。完成4个美丽乡村示范村建设，完成窄路加宽450公里、危桥改造30座、农村招呼站160个。104个村完成配电网改造。城市管理经验在全省推介。清水塘老工业区新关停企业15家，异地搬迁18家。湘江保护和治理“一号重点工程”第二个三年行动计划、两型社会建设第三阶段工作全面启动，城区洗水厂全部关停，霞湾港重金属污染治理等项目加速推进。

（四）凝聚合力推改革，发展活力持续释放。供给侧结构性改革扎实推进，“三去一降一补”取得实质性进展，71家烟花爆竹企业签订退出协议，5家煤矿企业关闭退出。城区商品住宅去化周期同比缩短2.8个月。大力开展“企业帮扶年”活动，交办各类问题2800余个，办结率达93.4%，企业帮扶经验被国务院推介。“营改增”等减税清费政策为企业和社会减负20亿元以上。政府存量债务置换和化解力度加大，综合债务率明显下降。加大“放管服”改革力度，下放市级管理权限29项，完成首批16家行业协会与行政机关脱钩试点验收。在全省率先下放城区私营企业注册登记权限，新增企业8500余家，增长38%。投融资体制改革深入推进，重组4家政府投资公司。财政资金统筹使用力度加大，财政管理绩效综合评价获评AA级。公共资源交易中心挂牌成立。基本完成农村土地承包经营权确权登记颁证工作。不动产统一登记在全市全面实施。公务用车、国企国资等领域改革有序推进。开放发展迈出坚实步伐。对外投资2.6亿美元，增长30%。保税物流中心（B型）、铁路口岸成功获批并加快建设。

（五）倾尽全力办实事，民生民利持续改善。民生支出持续增加，占财政总支出的73%。大力实施“七个一批”和“六大工程”，全面完成省脱贫攻坚考核任务。“民生100”工程成效显著，新增企业养老保险参保3.6万人，新增养老服务床位1371张。新增城镇就业5.2万人，新增农村劳动力转移就业1.58万人，农村五保对象基本医疗费全额减免。市属三所职业学校新校区开工建设。成功举办丙申年炎帝陵祭典、世界华人名人围棋邀请赛、首届株洲艺术节等活动。妥善处理市长热线和市长信箱来电来信共6万余件。建成治安岗亭60个，新增社会治安视频监控摄像头4800个，综治民调考评排名全省前列。防范和处置非法集资工作考核全省第一。安全生产和计生工作获评全省优秀。

各位代表，发展历程充满艰辛，发展成就鼓舞人心。这些成绩的取得，是市委正确领导的结果，是市人大、市政协监督与支持的结果，饱含了各位代表、各位委员的智慧和力量，凝聚了全市人民的心血和汗水。在此，我代表市人民政府，向全市广大干部群众，向人大代表、政协委员，向各民主党派、工商联、无党派人士和人民团体，向驻株部队官兵以及中央、省驻株单位，向离退休老同志，向所有关心支持株洲发展的各界朋友，表示崇高的敬意和衷心的感谢！

各位代表，成绩来之不易，经验弥足珍贵。我们深切地体会到：唯有坚持发展第一要务，强化产业支撑，突出项目攻坚，才能稳定经济增长、厚植发展优势。唯有坚持改革创新，突破发展障碍，才能增强动力、迸发活力。唯有坚持转型升级，加快转变发展方式，才能增创发展优势、实现逆势突围。唯有坚持发展为民，心中始终装着群众，才能筑牢执政根基、凝聚发展合力。唯有坚持从严从实，转变工作作风，才能做到真抓实干、干出实效。

我们必须清醒认识到，前进道路上还面临不少困难和问题。一是新旧动能转换艰难。一批支撑株洲发展的重化、冶炼等企业陆续退出，新动能短期内难以有效对冲旧动能下行的影响。二是投资增长后劲乏力。工业投资和民间投资不足，产业项目偏少，投资大、带动强、前景好的重大项目不多。三是社会治理任务较重。烟花爆竹、煤炭、危化等行业安全生产基础还不牢固，征地拆迁、非法集资等领域稳定风险防控、应急处置能力还需加强。四是改革攻坚难度较大。有的地区和部门推动改革的主动性、创造性还不够，改革红利难以有效释放。五是发展环境有待优化。政务服务效能有待进一步提高，行政事权下放、规范权力运行和推进政务公开的力度还需进一步加大，干部适应把握引领新常态的能力有待进一步提升。对此，我们要高度重视，采取有力措施加以解决。

二、未来五年形势和任务

未来五年，是株洲全面建成小康社会，进而开启基本现代化新征程的关键时期。迈向新征程面临新挑战，宏观形势依然错综复杂，经济新常态特征更加明显，经济下行和转型升级双重压力仍然较大。迈向新征程拥有新机遇，经济长期向好的基本面没有改变，株洲·中国动力谷建设步伐加快，新兴动能加速形成。地处“一带一部”和中部崛起的重要地带，战略地位持续提升，资源要素加速集聚，政策叠加效应更加彰显。迈向新征程肩负新使命，株洲作为长株潭核心增长极的重要组成部分，省委省政府寄予厚望，人民群众充满期待，我们必须以“核”的担当，展现“核”的作为。尤其是面对千帆竞发、百舸争流的区域竞争态势，不进则退，慢进也是退。“一万年太久，只争朝夕”。市第十二次党代会描绘了未来发展的宏伟蓝图，从新起点再出发，我们必须以时不我待的紧迫感，寝食难安的责任感，夙夜在公的使命感，大力弘扬奋勇争先、一往无前的“火车头精神”，全面实施“创新驱动，转型升级”总战略，先人一步，快人一拍，干在实处，走在前列，奋力谱写株洲发展新篇章。

今后五年政府工作的指导思想是：高举中国特色社会主义伟大旗帜，以马克思列宁主义、毛泽东思想、邓小平理论、“三个代表”重要思想和科学发展观为指导，深入贯彻党的十八大以来的各项方针政策和习近平总书记系列重要讲话精神，以五大发展理念为引领，认真落实省、市党代会精神，坚持创新引领、开放带动、两型发展、民生优先、文化提升，加快建成“一谷三区”，在全面建成小康社会和加快推进基本现代化进程中走在全省前列。

今后五年全市经济社会发展的主要奋斗目标是：主要经济指标增速高于全省平均水平，GDP力争达到4000亿元，一般公共预算收入力争达到500亿元，城乡居民收入增速高于GDP增速。按照“三年打基础、五年初见成效、十年左右实现基本现代化”的总体考虑，确保2017年完成整体脱贫攻坚任务、全面建成小康社会，继而全面开启基本现代化新征程，2020年完成“十三五”规划目标任务，2021年完成基本现代化的阶段性任务。

围绕上述指导思想和奋斗目标，突出抓好五个方面：

（一）坚持创新引领，加快建成株洲·中国动力谷。以建设国家自主创新示范区为契机，全力推进以科技创新为核心的全面创新。建设一批高端创新平台，培养一批创新人才，突破一批前沿技术和关键技术，培育一批创新型企业。围绕产业链布局创新链、打造服务链，促进“三链融合”，提升产业核心竞争力，加快建设创新型城市。深入对接“中国制造2025”“互联网+”战略，全面推进工业“1093”行动，实施“223”园区工程，加快建设制造强市。大力推进“中国动力谷十年行动计划”，全力打造“3+3+1”产业新体系，做大做强轨道交通、航空、汽车三大动力产业，加快建设一流的轨道交通装备制造基地、通用航空产业基地、新能源汽车基地，推动动力产业集群迈上4000亿元台阶，将株洲建设成为产业特色鲜明、发展动力强劲、享有国际声誉的动力之都。推进传统产业升级发展，打造中国瓷谷、服饰名城和烟花之乡。推进金融集聚区建设，努力打造湖南金融次中心，加快发展现代物流业，推动生产性服务业与制造业融合发展。

（二）坚持开放带动，加快建成“一带一部”开放发展先行区。坚持全域开放，构建开放型经济新体制，培育全面开放竞争新优势。加快建立集铁路、公路、水运、空运为一体的综合性、立体化开放大通道，实现与“一带一路”互联互通，构建开放大平台，加快发展口岸经济。坚持“引进来”，实施引资引技引智升级行动，瞄准产业链前端、价值链高端引进企业和项目，大力承接产业转移，打造具有核心竞争力的产业集群。坚持“走出去”，支持企业扩大对外投资、开展海外并购和承包工程项目，以产品走出去带动产能走出去，增强轨道交通、航空、汽车、硬质合金、陶瓷、烟花等优势产业的国际竞争力。坚持以开放带改革、以改革促开放，重点推进行政管理体制、市场主体、财税金融体制等改革，努力打造效率高、服务优、成本低、口碑好的营商环境。

（三）坚持两型发展，加快建成老工业城市转型发展示范区。牢固树立“绿水青山就是金山银山”的理念，深入推进两型社会建设第三阶段工作，大力实施“碧水蓝天青山净土”行动，突出抓好“两保两治一示范”，到2020年基本建成两型社会。突出生态保护，实施绿心保护提升示范工程、绿色生态屏障工程、旅游区保护与建设工程、江河湖库水系连通工程，加强山水林田湖的生态修复与保护。突出制度保障，加快生态文明体制改革，形成源头预防、过程控制、责任追究的生态文明制度体系。按照“三年大见成效，五年完成综合治理”的目标，全面完成清水塘老工业区搬迁改造和综合治理，打造老工业区生态修复、工业遗址利用、绿色产业高地三张名片。大力推进湘江保护和治理“一号重点工程”，加强“一江两水四港”整治，确保湘江流域水质不断好转。大力开展两型标准建设示范工程，积极倡导绿色生产生活方式，让绿色发展成为全社会的行动自觉、价值追求和精神风尚。

（四）坚持民生优先，加快建成城乡统筹发展幸福区。全力打赢脱贫攻坚战，加强脱贫后续政策扶持，确保全市人民同步实现小康，决不让一个群众在小康路上掉队。加快推进以人为核心的新型城镇化，大力构建新型城镇空间体系，按照“一核一圈一廊”空间布局，加快“北联、东拓、南进”步伐，优化空间形态。深入实施交通畅通、旧城提质、城市绿荫、碧水蓝天“四大行动”，积极推进智慧城市、海绵城市建设，提升城镇品质，打造更高水平的生态宜居城市。加快构建新型城镇产业体系，推进优势产能向县域转移布局，共同打造产业链和产业集群。以农业产业升级“十百千”工程、农产品加工千亿产业培育工程等为抓手，走出“一产接二连三”的互动型、融合型发展新路。加快构建新型城镇公共服务体系，完善基本养老、基本医疗和失业、工伤、生育等保险制度，基本实现全覆盖。大力建设教育强市、健康株洲和平安株洲，让人民群众在共建共享中有更多的获得感。

（五）坚持文化提升，努力打造更具魅力和影响的株洲形象。以建设国家公共文化服务体系示范区为契机，大力发展文化事业，实施“六大文化惠民”工程，推进基本公共文化服务标准化均等化。以重大文化项目建设为抓手，加强文化与体育、旅游、科技深度融合，大力发展文化创意产业。推动工业文化遗产保护地方立法，深入挖掘炎帝文化、红色文化、书院文化等特色资源，形成“一带一轴”“一县一品”特色文化产业格局，提升文化核心竞争力，打造富有张力、充满活力、独具魅力的人文城市。以“文明提升十大行动”为重点，深入开展中国梦和社会主义核心价值观学习教育，推进公民思想道德建设工程，巩固文明创建成果，加大县乡文明创建力度，提升社会文明程度。促进对外文化交流，加大株洲形象的策划、宣传和推介力度，传播株洲声音，扩大株洲影响，展现“神农福地、动力之都”的独特魅力，进一步提升株洲的知名度和美誉度，增强全市人民的自豪感和归属感。

三、2017年工作安排

2017年是党的十九大召开之年，是贯彻落实市第十二次党代会精神的首战之年，是脱贫攻坚、全面建成小康社会的决胜之年，是供给侧结构性改革的深化之年。我们要全面贯彻中央、省委、市委经济工作会议精神，坚持稳中求进、进中争先工作总基调，以新发展理念为指引，以提高发展质量和效益为中心，以供给侧结构性改革为主线，持续开展“项目攻坚年”和“企业帮扶年”活动，全面做好稳增长、促改革、调结构、惠民生、防风险各项工作，推动经济平稳健康发展和社会和谐稳定，确保全市整体脱贫，奋力夺取全面小康社会新胜利，以优异成绩迎接党的十九大胜利召开。

全市经济社会发展的主要预期目标是：GDP增长8.5%左右，固定资产投资增长16%，规模工业增加值增长8%，社会消费品零售总额增长12.5%，一般公共预算收入增长7%，城乡居民收入增长高于经济增长速度，万元GDP能耗完成省下达任务。完成今年的目标任务，重点抓好以下五个方面的工作：

（一）突出供给侧结构性改革，着力激活发展新动力

继续抓好“三去一降一补”。去产能，重点推进株冶等企业转移转型，完成4家中央省属企业和所有中小冶炼化工企业关停搬迁，腾退收储土地2000亩以上，确保清水塘老工业区搬迁改造取得标志性、突破性进展。关闭50家烟花爆竹企业和5家以上煤矿企业。加大“僵尸企业”处置力度，妥善做好企业债务化解和人员安置工作。去库存，重点深化以购租并举为方向的住房制度改革，合理调控土地供应，提高棚改货币化安置率，加大非住宅领域去库存力度，促进房地产市场健康平稳发展。去杠杆，重点降低政府平台公司负债水平。防范和打击非法集资，坚守金融安全底线。降成本，重点开展“企业帮扶年”活动，完善帮扶机制，落实正税清费政策措施，有效降低制度性交易成本，降低水电气、融资、物流等生产成本。补短板，聚焦第三产业、非公经济、开放型经济、县域经济等领域，实施一批补短板工程。

推进重点领域改革。深化“放管服”改革，继续推进项目建设审批制度改革，下放一批审批权限，清理规范行政审批中介服务，积极开展“减证便民”行动。探索市、区财政事权和支出责任划分改革，扩大和细化财政预决算公开，加强财政资金统筹整合和专项资金绩效评估。完善综合治税信息管理平台。推进国有企业混合所有制改革试点，完成市级政府平台公司改革重组。组建金融服务集团，全面完成农信社改革工作。完成农村土地承包经营权确权登记颁证工作，启动承包土地“三权分置”以及农村土地征收改革，争取进入集体经营性建设用地入市、宅基地制度改革试点。推进全国水生态文明建设及河湖管护体制机制创新试点，全面实行“河长制”。

加快发展非公经济。抓紧制定市场准入负面清单，出台民间投资引导目录，促进民营企业特别是中小微企业公平参与市场竞争。推广PPP模式，引导更多社会资本进入基础产业、基础设施、市政公用事业、保障性住房、金融服务等领域。积极探索“多证合一”，推进工商登记便利化，激发市场主体活力。依法平等保护非公有制经济产权，营造公平、公正、透明、稳定的发展环境，促进各类企业各展其长、共同发展。

提升开放发展水平。统筹规划建设水运港口、铁路口岸和保税于一体的进出口共享平台，建成保税物流中心（B型）。深化关检合作“三个一”，加快推进通关一体化。争取渝长厦高铁过境株洲、“湘欧快线”延伸至株洲。完善外贸奖补激励政策，提高中小企业出口占比，全年进出口增长10%以上。着力推动优势产品出海，积极参与国际展会，提高株洲产品的知名度和国际议价能力。

（二）突出产业转型，着力发展实体经济

大力推进科技创新。增强创新供给能力，为实体产业发展提供源动力。加快国家自主创新示范区建设，全面落实“五张需求清单”，新认定高新技术企业20家以上。大力实施制造业创新能力建设专项行动，争取国家级轨道交通制造业创新中心落户株洲，力争100个以上项目进入国家省级科技计划。实施100项科技成果转化工程，加快科技成果转化基地建设，实现高新技术产业增加值增长15%以上。推进“双创”建设专项行动，新增众创空间10家以上，科技企业孵化器运营面积达10万平方米。实施院士培育工程和“万名人才计划”，积极与高校、科研院所、企业开展战略合作，建好湖南省协同创新研究院株洲分院，拓展创新空间，推动产业发展。

做大做强制造业。坚持把建设制造强市作为振兴实体经济的重要抓手，大力实施工业“1093”行动，全力打造株洲·中国动力谷，培育制造业新的增长点。加快全国质量强市示范城市创建，推进国家质量技术检验检测中心、国家出口日用陶瓷电瓷质量安全示范区建设。开展质量提升行动，弘扬“工匠精神”，扩大高质量产品和服务供给，推动株洲产品向株洲品牌转变。推进智能制造，重点在三大动力产业开展智能工厂和数字化车间建设，创建智能制造示范企业2个以上、示范车间4个以上、示范项目5个以上。按照建链、补链和强链思路，大力开展引进100家配套企业活动，发展壮大配套产业。持续实施工业“十百千”工程，新增规模工业企业100家以上。全面落实“一权两制一司”管理体制和“六个一”工作模式，推动要素向园区集聚、项目向园区集结、产业向园区集中。建成标准厂房200万平方米以上，确保园区新开工工业项目100个以上。推进国家级经济开发区创建，完成渌口、醴陵经济开发区扩区，提升园区承载能力。

持续提升现代服务业。积极申报国家服务业综合改革试点，加快推进九大服务业集聚区建设，力争全年服务业增加值达到1000亿元以上。继续实施“1511”工程，提升传统商贸业。大力发展现代物流、健康养老、信息消费、物联网等产业，培育新的消费热点。加快发展会展经济，打造轨道交通产业国际峰会、航空产业国际峰会、新能源汽车产业峰会、芦淞服饰文化节、醴陵陶瓷博览会等五张名片。迎接大众旅游时代，开发旅游黄金线路和特色商品，发展工业游、自驾游、生态休闲游等旅游业态，推动醴陵市、炎陵县国家全域旅游示范区建设，加快酒埠江、云阳山、大京等旅游项目综合开发，创建炎帝陵国家5A级景区。

加快发展现代农业。深入推进农业供给侧结构性改革，培育农业发展新动能。加强高标准农田建设，实施粮食高产工程，确保粮食播种面积稳定在380万亩以上，蔬菜种植面积稳定在100万亩以上。加大云龙花卉、攸县麻鸭、茶陵黄牛、炎陵黄桃等品牌创建和产业发展力度。大力发展生态农业、休闲农业，启动出口食品农产品示范区建设，增加绿色农产品供给，着力建设优质农副产品基地。依法规范土地流转和托管，发展适度规模经营，培育新型农业经营主体，推进农业产业升级“十百千”工程，积极争取7个现代农业特色产业园列入省项目笼子。加快推进市农副产品批发交易中心建设。加大先进农机装备推广力度，重点推进水稻生产全程机械化。坚守农业农村工作底线，切实抓好防汛抗旱、森林防火、动植物防疫和农产品质量安全监管工作。

（三）突出项目攻坚，着力扩大有效投资

加大项目争引力度。盯紧国省投资重点、重大工程包、专项债券等政策，策划开发重点前期项目200个以上。依托“港洽周”等招商活动平台，对接“万商入湘”专项行动，围绕产业集群、总部经济、研发中心等重点，大力开展点对点、产业链、专业化招商，着重引进一批带动性强、成长性好、关联度高的大项目和大企业。力争全年国家级园区引进1个过20亿元的产业项目，省级园区引进1个过10亿元的产业项目。严格招商引资考核，确保重点招商签约项目履约率达70%以上。

加快项目建设进度。深入开展“项目攻坚年”活动，全年实施重点项目300个以上，完成投资1000亿元以上，新开工建设200个、竣工100个。制造业方面，启动608所及南方公司科研生产能力提升、IGBT产业化二期建设，加快轨道装备产业扩能与智能制造建设，建成北汽二工厂。现代服务业方面，启动铁路国际物流园建设，加快湖南移动数据中心、酒埠江综合开发、汽车博览园建设。现代农业方面，加快恒新林业农产品原料基地及深加工产业园项目进度，建成龙华龙牧二期。交通建设方面，启动长株攸快速通道、醴娄高速和S105旅游通道建设，加快东城大道、莲易高速、湘江大道建设。完成农村公路窄路加宽226公里、安保设施建设220公里，实施危桥改造、渡改桥建设17座，建成农村客运招呼站100个。园区建设方面，启动清水塘疏港大道、物流西路建设，加快航空城机场大道、千亿大道建设，基本建成轨道交通城中车大道、田心大道。城市建设方面，加快“一站一路”项目建设，启动株洲火车站改造，基本建成铁东路核心段并启动北段建设。推进中环贯通工程建设，加快北环线及石峰大桥、建宁大桥匝道建设。启动市区有轨公共交通示范线建设。推进湘江河东段综合治理工程，建成河东湘江风光带，启动枫溪段建设。启动龙母河片区综合开发项目建设。

加强要素保障强度。拓宽项目融资渠道，加大政策性、开发性、商业性金融资金争取力度，新增社会融资400亿元以上。加强产业专项建设基金申报，发起设立产业发展基金和城市发展基金。严格管理政府投资项目，提高投资效益。全力保障项目用地需求，大力推进城乡建设用地增减挂钩工作，深入开展“清零行动”和集中拆违控违攻坚大行动，确保全年完成集体土地征地拆迁3.2万亩、国有土地房屋征收42万平方米、城市拆违100万平方米。畅通项目审批“绿色通道”，从严打击强揽工程、恶意阻工等违法行为，优化项目建设环境。

（四）突出城镇提质，着力建设宜居家园

实施“四大行动”，提升中心城区品质。大力实施交通畅通行动。拉通神农大道、天池路等一批骨干道路，打通新塘路、黄山西路等一批城市“断头路”，疏通响石广场、珠江路与泰山路交叉口等一批交通堵点，修通及改造湘运路、劳动路等一批支路和小街小巷，优化城市路网，提高通行能力。加快“公交都市”创建，启动城乡公交试点，建设市区快速公交示范线，深化出租车行业改革，规范网约出租车监管，让市民出行更加方便。大力实施旧城提质行动。积极推进中心城区企业“退二进三”“退城入园”，疏解中心城区功能。加快实施一批棚户区、老旧小区、城中村、历史老街区等改造项目，推动老城区有机更新。统筹开展海绵城市、地下综合管廊、“两供两治”建设，完善城市综合配套功能。大力实施城市绿荫行动。推进“六大工程”建设，基本建成神农绿轴、中环大道节点景观，开工建设枫溪山、新苗、凤凰山、雪峰岭、云峰森林植物园等城市公园，着力编织城市绿网、增加城市绿量。大力实施碧水蓝天行动。继续实施湘江保护和治理“一号重点工程”，重点推进清水塘综合治理，完成第三方治理试点任务，推进世行贷款项目实施，启动搬迁企业场地污染整治。全力整治城市黑臭水体，加快枫溪污水处理厂、河西污水处理厂、云龙水质净化中心以及配套管网建设改造。综合治理城市扬尘，加大黄标车淘汰力度，大力推广应用新能源汽车，改善大气质量。

推进全域统筹，促进城乡一体发展。加快株洲县撤县设区、攸县撤县设市和茶陵县省直管县改革。完成“一区一县九镇”试点地区“多规合一”工作，分层次有重点开展城市设计。实施城区“城市修补、生态修复”和农村“危房改造、环境改善”。提质改造县乡道路，完成全市贫困村主干道硬化。推进“五小”水利建设，抓好饮用水应急水源建设前期工作。加快城乡宽带、广播电视网络等信息基础设施建设，实现通讯信号全域覆盖，加快光纤入户步伐。启动安炎天然气管道建设。引导城区优势产业、骨干企业向县域延伸产业链、布局新项目，鼓励县域产业园区承接符合环保要求的清水塘老工业区企业绿色搬迁。重点打造20个特色功能镇。严格落实《株洲市农村村庄规划建设管理条例》，加快实施美丽乡村提质升级行动，抓好“一廊十片百村”示范工程，重点创建50个美丽乡村。

加强“四化”管理，推动城镇管理升级。运用市场化、标准化、信息化、法治化手段，推动城市管理向城市治理转变。深化城市管理和综合执法体制改革，整合部门职能，优化一线执法力量。加大政府购买服务力度，吸引社会力量和社会资本参与城市管理，启动城市垃圾分类试点工作。完善社区公共服务设施，推动城市管理向小区延伸，打造方便快捷生活圈。畅通公众有序参与城市管理渠道，倡导城市管理志愿服务。提升市民文明素养，巩固文明创建成果，确保蝉联全国文明城市。

（五）突出小康决胜，着力改善民生福祉

坚决打赢脱贫攻坚战。将脱贫攻坚作为头等大事和第一民生工程来抓，紧扣“两不愁、三保障”目标，落实扶贫政策，加大扶贫投入，强化金融支撑，严格督察考核，大力激发贫困地区、贫困群众脱贫攻坚的内生动力。全力推进精准脱贫“七个一批”、基础设施扶贫“六大工程”、城市支持农村“四大基地”建设，确保两个贫困县摘帽、贫困村退出、贫困人口脱贫。坚持推进脱贫与小康齐头并进，强化问题导向，严格对照全面小康考核指标体系，找出薄弱环节，逐项落实措施，确保五县市同步实现全面小康，奋力夺取全面小康社会新胜利。

深入实施“民生100”工程。坚持以人民为中心的发展思想，深入实施“民生100”工程，让发展的每一步都踏准人民的需求。在全面完成各项民生指标任务的同时，从解决好人民群众普遍关心的突出问题入手，重点办好以下实事：改造城市棚户区2.6万户、农村危房6300户，着力解决住房难题；改造城区60条支路和小街小巷，打通群众出行“最后一百米”；实施5处城市黑臭水体综合治理，促进城市水环境持续改善；解决12万农村人口安全饮水问题；建设农产品追溯系统追溯点5个，五县（市）全部建成病死畜禽无害化处理中心，保障农产品安全；建成公共停车场6个，疏通交通堵点4个，规范中小学周边交通秩序管理，缓解城市停车、行车难题；开展居民小区餐饮油烟、违法违规搭建、占道经营和夜间噪声污染综合治理，规范居民小区管理秩序；推进10个老旧小区、27个农贸市场综合整治改造升级；优化城乡学校布局，新增学位2万个，缓解就学难题。

不断提高社会保障水平。全面落实“六补贴两补助”等就业创业政策，着力完善零就业家庭、残疾人、低保对象等困难群体的就业援助制度，确保全年新增城镇就业4.8万人、农村劳动力转移就业1万人。推进社保扩面提标，完成全民参保登记计划，新增企业养老保险参保3万人。提高城乡低保标准，实现农村低保标准与贫困标准融合。开展“救急难”工作试点，健全社会救助体系。加快爱心大院三期建设。加快完善社会养老保障体系，深化机关事业单位养老保险制度改革。推进城乡居民医保政策整合和大病保险市级统筹。加快市民“一卡通”建设。完善住房保障体系和公积金制度，切实改善城乡居民居住条件。

全面推进社会事业发展。加快建设教育强市，加强普惠性民办幼儿园建设管理，完成200所义务教育学校和教学点的标准化建设，推进普通高中高品质学校建设，完成长郡云龙学校主体工程，实现市特殊教育学校秋季入学，加快铁道职院、中医高专和市属三所职业院校搬迁建设，支持湖南工业大学建设高水平综合性大学，共建醴陵陶瓷学院。大力推进健康株洲建设，深化市中心医院与湘雅医院的医疗合作，推进云龙医院建设和田心医院提质改造，加快区域医疗中心和远程会诊系统建设，实施分级诊疗制度，提升基层医疗服务水平。以公立医院改革为重点，深化医药卫生体制改革，理顺医药价格，鼓励社会办医。坚持计划生育基本国策，深化计生服务管理改革。建成市青少年活动中心、市妇女儿童活动中心、市老年活动中心，启动市图书馆建设，完成市博物馆、美术馆搬迁和市群艺馆、戏剧传承中心提质改造。确保创建国家公共文化服务体系示范区迎检达标。精心筹办第十九届中国老年合唱节、全球华人羽毛球比赛、市第十三届运动会、丁酉年炎帝陵祭祖典礼等重大活动。

全力维护社会和谐稳定。加强社会治安综合治理创新，全面推行网格化管理、社会化服务和信息化支撑，夯实社会治理基础。完善社会治安立体防控体系，巩固“平安株洲”创建成果。深入开展“七五”普法，全面推进法治株洲建设。加大信访积案化解力度，组织开展“信访维稳百日竞赛”活动，有效化解社会矛盾纠纷。依法管理民族宗教事务，确保民族宗教工作大局平稳有序。加快推进安全生产领域改革，严格落实安全生产责任制，严防重特大事故发生。开展食品药品安全专项整治，严格市场监测和准入，创建国家食品安全示范城市。加强国家安全、国防动员、国防教育、人民防空、双拥共建和优抚安置工作，支持驻株部队建设，推进军民融合发展。

四、切实加强政府自身建设

肩负历史使命、承担发展重任、顺应人民期待，我们必须加强自身建设，提高治理能力，着力建设“法治、诚信、创新、效能、廉洁”政府。

（一）依法行政，建设法治政府。严格落实《法治政府建设实施纲要》，把政府活动全面纳入法治化轨道。适时调整公布政府权力、责任、收费“三个清单”，依照法定权限、规则和程序行使权力，形成清晰的政府权责边界。不断健全市政府“3+4”决策体系，提高依法决策水平。主动接受人大及其常委会的法律监督、工作监督，自觉接受政协的民主监督，认真办理人大代表建议和政协委员提案，广泛听取各民主党派、工商联、无党派人士和人民团体的意见建议。

（二）守信重诺，建设诚信政府。建立政府守信践诺机制，健全政务诚信评价体系，强化对发展目标落实、招商履约落地、为民办实事工作的绩效考核，以“徙木立信”之态取信于民。加大政务公开力度，完善政府诚信约束和问责机制，进一步提高政府公信力。建立统一的信用信息征信平台，健全社会信用评估体系，建立守信奖惩机制，形成全社会的“信任共同体”，让诚实守信成为全民的共同价值追求。

（三）开拓进取，建设创新政府。坚决冲破妨碍发展的思想观念，革除束缚发展的体制弊端，大力推进方法创新、管理创新、政策创新。重视发挥智库作用，引入重大政策措施第三方评估。创新公共服务提供方式，完善政府购买服务清单，提高公共服务供给质量和效率。加快“智慧株洲”建设，打造网上无纸化行政审批服务平台，推行“互联网+”政务服务，整合大数据，构建全方位、全覆盖、全过程的政务服务体系，让信息多跑路、群众少跑腿。

（四）务实担当，建设效能政府。强化担当意识，面对困难不退缩、面对矛盾不回避、面对责任不推诿，全力以赴为发展解难、为群众纾困。强化绩效考核，加强政务督查，严格行政问责，打通政策措施执行的“最后一公里”，确保政令畅通。深入开展“马上就办、真抓实干”活动，大力推行“五问工作法”，推进政府工作项目化和责任化，提高执行力和落实力。贯彻落实“三个区分开来”的要求，健全激励机制和容错纠错机制，旗帜鲜明地为敢于担当者担当，为敢于负责者负责。

（五）从严从实，建设廉洁政府。自觉履行“两个责任”，严格落实党风廉洁建设各项规定，严惩各类不正之风。加强和规范党内政治生活，不断增强政府工作人员自我净化、自我完善、自我革新、自我提高能力。加强权力运行监督，做到有权必有责、用权必担责、滥权必追责。健全土地交易、招投标、政府采购等重点领域的反腐机制，加强对财政资金、重大项目的审计监察，从源头上遏制腐败，让暗箱操作没有空间，让腐败行为无处藏身。

各位代表，历史的画卷，总是在砥砺前行中展开；时代的鼓点，总是在克难奋进中擂响。站在全面建成小康社会、加快实现基本现代化的新起点、新方位，置身推动株洲改革发展的新时空、新坐标，我们要把发展的眼光放得更远，把奋斗的脚步踩得更实，不忘“为山九仞”的风险，保持“久久为功”的韧劲，拿出“舍我其谁”的气概，用担当点亮希望之光，以进取实现心中梦想。

各位代表，“雄关漫道真如铁，而今迈步从头越”。让我们更加紧密地团结在以习近平同志为核心的党中央周围，在省委、省政府和市委的坚强领导下，不忘初心，继续前进，为加快建成“一谷三区”、实现“两个走在前列”的宏伟目标而努力奋斗！
